# MESOTHELIOMA V/S ADENOCARCINOMA OF LUNG DO SERUM MARKERS HELP?

**DOI:** 10.4103/0970-2113.45287

**Published:** 2008

**Authors:** Neeraj Gupta

**Affiliations:** Assistant Professor, Deptt of Respiratory Diseases & Tuberculosis JLN Medical College, Ajmer (Raj.) 305001

Going through an enlightening case report of ‘25 year old male with pleural thickening’ by Abhilasha Ahuja et al^1^, the matter of differences between adenocarcinoma of lung & malignant mesothelioma raise many debates.

The diagnosis of malignant mesotheliomas is usually established by a combination of histology & immunohistochemical stains without-resort to electron microscopic examination. However, electron microscopy plays a decisive role in cases with unusual morphology, anomalous histochemical or immuno-histochemical reactions^2^.

Apart from the differences, as described by the authors, between adeno-carcinoma of the lung and mesothelioma, the role of serum markers need further mention. As it may be a case with any cancer in the body, sensitive markers can facilitate early diagnosis and thus early therapeutic intervention.

Soluble Mesothelin Related Protein (SMR) is one such maker which has been studied in Malignant Mesothelioma of pleura and other malignant & non malignant lung and pleural-diseases^3^.

The authors opined that a raised serum SMR concentration is indicative of mesotheliomas with sensitivity of 84% and a specificity of 100%. Other non-mesothelioma tumors that can also cause increased SMR concentrations are ovarian, lung and breast carcinomas, so specificity in studies that include such patients will definitely fall below 100% as was in Scholler and collague's study.^4^

However, one of the salient feature of study by Robinson et al^3^ was that none of the seven patients with lung adenocarcinoma in their study had raised SMR concentrations suggesting that this assay might be useful to distinguish the two histologically almost similar conditions.

Moreover, the levels of SMR are related to tumor load. Significantly higher levels were found when the maximum pleural tumor width was more than 3 cm. Higher SMR levels in asbestos exposed individuals also might be a predictor of disease development or a sensitive marker of early disease in some patients^4^.

SMR level estimation is probably the first blood test that is commercially available & may prove an exiting step towards better diagnosis of Mesothelioma and probably to differentiate from adenocarcinoma of lung^5^.
